# Body Composition Profile of Young Patients With Phenylketonuria and Mild Hyperphenylalaninemia

**DOI:** 10.5812/ijem.16061

**Published:** 2014-07-01

**Authors:** Artemis Doulgeraki, Astrinia Skarpalezou, Areti Theodosiadou, Ioannis Monopolis, Kleopatra Schulpis

**Affiliations:** 1 Department of Bone and Mineral Metabolism, Institute of Child Health, Athens, Greece; 2 Department of Inborn Errors of Metabolism, Institute of Child Health, Athens, Greece; 3 Department of Radiology, Children’s Hospital, Athens, Greece; 4 Department of Biostatistics, Institute of Child Health, Athens, Greece

**Keywords:** Muscle, Bone, Phenylalanine, Children

## Abstract

**Background:**

There is evidence in support of low bone density in young patients with disorders of phenylalanine metabolism; however, little is known about muscle and fat mass in these patients, especially in those with mild hyperphenylalaninemia (mHPA).

**Objectives:**

We aimed to evaluate body composition of children and adolescents with early-diagnosed disorders of phenylalanine metabolism.

**Patients and Methods:**

The study was conducted in the Institute of Child Health, which is the national center that performs newborn screening. Bone, muscle, and fat mass of 48 patients with phenylketonuria (PKU) and 32 patients with mild mHPA, aged five to 18 years, were compared to 57 age- and sex-matched controls. Dual energy X-ray absorptiometry was used for this purpose.

**Results:**

Compared to controls, bone mineral density (BMD) was lower in patients with PKU (mean total body BMD z score, 0.11; P = 0.03) and in those with mild mHPA (mean lumbar BMD z score, -0.34; P = 0.01). Lean body mass and fat mass were not significantly affected in the study group. Comparison between the two patients groups did not reveal any difference in body composition profiles; however, pubertal status appeared important for within-group comparisons. Fat mass was significantly increased in teenagers with PKU, which was more evident in those with poor dietary compliance irrespective of gender (fat mass z score, 0.66; P = 0.018). Finally, positive correlations were found not only between bone, muscle, and fat mass in both groups, but also between fat mass and Phenylalanine levels in patients with PKU (r, 0.46; P = 0.001).

**Conclusions:**

Bone mineral density appears suboptimal in young patients with disorders of phenylalanine metabolism. Adolescents seemed more prone to obesity, especially when their dietary adherence was poor, whereas muscle mass was not considerably affected. To ensure healthier bones and less fat content, close follow-up as well as proper lifestyle advice is needed.

## 1. Background

Primary hyperphenylalaninemia (HPA) is the most common inborn error of protein metabolism with an incidence of one in 10000 newborns(1), which is similar to that found in the Greek population (Dr KH Schulpis, personal communication. It results from the functional impairment of the phenylalanine hydroxylase (EC 1.14.16.1) enzyme, which in return leads to toxic phenylalanine (Phe) levels. Classic phenylketonuria (PKU; OMIM 261600) and mild hyperphenylalaninemia (mHPA) represent the two ends of the disease spectrum, with the former being the most severe one. They are autosomal recessive disorders. Fortunately, prompt detection of patients is possible through neonatal screening, which is established in most parts of the world including Greece. The diagnosis of PKU is established when Phe levels exceed 1080 μmol/L on a regular diet, whereas in mHPA, Phe levels range between 360 and 600 μmol/L (reference Phe value < 168 μmol/L). This classification reflects the residual enzymatic activity and can be confirmed with molecular analysis (2). Classic PKU is a devastating disease if left untreated; it causes mental retardation, seizures, behavioral disturbances, and eczema. A lifelong special diet to control the disorder with the replacement of natural protein (eg, dairy products) with Phe-free amino acid supplements as well as vitamins and trace minerals is require. Dietary compliance is essential to prevent complications (3). For patients with mHPA, no dietary intervention is necessary if they are older than five years (4). For younger patients, there is much debate on whether they should be treated. In general, patients with mHPA maintain a nontoxic Phe level (< 600 μmol/L) while on a balanced nutrition regimen. Numerous studies report poor bone health in PKU due to lack of dairy products consumption and the disorder itself (5); however, studies on muscle and fat masses (MM and FM, respectively) are scarce with conflicting results. To our knowledge and as far as mHPA is concerned, there was no studies on body composition using dual-energy X-ray absorptiometry (DXA).

## 2. Objectives

The aim of this cross-sectional study was to evaluate body composition of patients with PKU and mHPA using DXA, which is considered as the gold standard of diagnosis, as it employs a three-compartment model: bone mass, MM, and FM (6). The incentive for the study was the clinical impression that many adolescent with PKU and poor dietary adherence tended to appear overweight or obese. This study was an effort towards improving everyday practice and proper lifestyle counseling to ensure better quality of life for these patients.

## 3. Patients and Methods

### 3.1. Study Population

The study was conducted over a two-year period at the Institute of Child Health in Athens, the national center for newborn screening in Greece. The participants were 80 Caucasian patients with early-diagnosed (neonates; age range, 7-10 days old) disorders of Phe metabolism (43 girls and 37 boys; mean age, 10.88 ± 3.47 years). Of those, 48 were diagnosed with PKU (23 girls and 25 boys; mean age, 10.9 ± 3.43 years) and 32 with mHPA (14 girls and 18 boys; mean age, 10.85 ± 3.6 years). Their classification was based on the initial Phe value obtained through neonatal screening (Guthrie card, enzymatic assay), which were Phe > 1080 μmol/L for PKU and Phe 360 to 600 μmol/L for mHPA. It was also confirmed by molecular analysis. Dietary treatment had been initiated within the first two weeks of life. At the time of the study, the daily protein intake of patients younger than eight years of age with PKU was replaced largely by PKU2 Prima (Milupa AG). Older patients received PKU2 Secunda (Milupa AG). These are Phe-free amino acid mixtures enriched with vitamins and trace minerals. Good dietary compliance was evident by mean annual Phe levels of 120 to 360 μmol/L for children younger than 12 years of age and 120 to 600 μmol/L for those older than 12 years of age. In contrary to the patients with PKU, patients with mHPA were on a free diet during the study period, in accordance to Greek guidelines for children older than five years of age and their nutrients intake met the recommended daily allowances values. All patients were followed up regularly (every 6-12 months) and their Phe concentration was monitored closely (every 1-2 months). They also reported unrestricted participation at school exercise programs. 

Exclusion criteria were the following: late diagnosis (beyond the neonatal period), toddlers and adults (the available DXA software was suitable for those with 5-18 years of age), history of recent fracture (within three months preceding the study), positive family history for osteoporosis, and the presence of other chronic conditions or taking medications that could alter bone metabolism or growth. On the day of DXA, the recruited patients’ anthropometric values were measured, ie, height (Ht [cm]; using Harpenden stadiometer) and weight (Wt [kg]; using calibrated electronic scale). In addition, body mass index (BMI [kg/m^2^]; Wt divided by Ht square) was calculated. The obtained values were subsequently plotted on the Greek growth charts (7, 8). Pubertal stage evaluation with the Tanner and Whitehouse method was performed clinically. Stages 1 and 2 were classified as prepubertal and stages 3, 4, and 5 as pubertal.

### 3.2. Body Composition Analysis

Bone mineral content, which is the absolute value of bone mass (BMC [g]), lean tissue mass (LTM [g]), and FM (g) were derived by total body (TB) DXA, using a GE Lunar Prodigy apparatus enCore, 2008, USA with pediatric software (pediatric Italian reference population). Before scanning, a thorough calibration procedure was performed on a daily basis according to the manufacturer’s protocol. All scans were performed by the same qualified technician. Precision was expressed automatically with a coefficient of variation, which was 1.5% for spine bone mineral density (BMD) and 1.1% for TB BMD. Areal BMD was measured (g/cm²) at the lumbar spine (LS; L1-L4 spines) and TB. The results were automatically converted to z scores by the apparatus. Z scores were also calculated for LTM and FM values to allow comparisons between different age groups.

#### 3.2.1. Controls

As there was no Greek reference database for body composition analysis, 57 age- and sex-matched Caucasian controls were evaluated using DXA. They were healthy Greek children and adolescents with normal growth (those with Ht or Wt < 5 ^th^ or > 95 ^th^ percentile were excluded) receiving no supplements or medications. Their absolute values of all body composition parameters were used as a database for Z score calculations.

#### 3.2.2. Laboratory Investigations

On the day of body composition assessment, fasting serum samples were collected for routine biochemistry using standard techniques (in patients groups only). The determined parameters were: calcium, magnesium, phosphate, alkaline phosphatase, total protein, albumin, and creatinine. Additionally, Phe level was determined with the Biotronic LC 5010 aminoacid autoanalyzer, Beckman Instruments Inc., Fullerton, CA, USA). The mean value of Phe levels during the year before the study was also estimated in retrospect.

### 3.3. Statistical Analysis

Descriptive statistics were performed in all groups (PKU, mHPA, and control groups) and were presented as mean ± standard deviation (SD). All absolute anthropometric and body composition values were converted to z scores with the following equation: Z score = (actual value- mean value for age and sex)/SD.

Z score values between -2 and +2 were considered normal (9), whereas values between -1 and -2 were determined as low-normal. To check the normality of the data distribution, Kolmogorov-Smirnoff test was used. For comparison of numerical parameters between two different groups, a student’s t test was performed. Statistical analysis was done in three stages; initially, PKU and mHPA were compared separately to the control group. Then, a comparison was made between PKU and mHPA groups. Finally, within-group comparisons were studied, ie, boys vs. girls and prepubertal vs. pubertal individuals. To determine any possible associations between different body composition parameters in each patients group, Pearson’s correlation was employed. The level of statistical significance was set at P < 0.05. For data analysis, SPSS v. 17.0 (SPSS Inc., Chicago, IL, USA) was used.

### 3.4. Ethical Approval

The Ethics Committee of the Institute of Child Health approved this descriptive study. Informed consent was obtained from all participants (including controls) and their guardians. 

## 4. Results

### 4.1. Study Population Profile

The anthropometric characteristics of the patients groups are summarized in Table 1. They are expressed in z scores. In addition, data regarding age, sex, pubertal stage, and annual mean Phe levels are presented (Table 1). Evaluation of growth parameters (Ht, Wt, and BMI) by the Greek growth charts revealed increased Wt and BMI in patients with PKU, whereas no significant difference was found between patients with mHPA and their healthy counterparts. Fracture history was unremarkable in both groups of patients and they had no complaints of musculoskeletal pain.

### 4.2. Laboratory Investigations

All measured indices were within normal limits. Mean Phe levels during the preceding year of study are presented in Table 1. Based on these values, 27 (56%) out of 48 patients with PKU showed good dietary compliance.

### 4.3. Body Composition Parameters

In comparison to controls, patients with PKU had lower TB BMD Z scores (mean, 0.11 ± 1.1; P = 0.03). Among 48 patients with PKU, there were only one with low (Z score at both sites < -2) and eight with low-normal BMD (-2 ≤ Z scores ≤ -1). No significant difference in LTM or FM was found between patients with PKU and their controls. Correction for height, where appropriate, yielded similar results. 

The patients with mHPA had lower BMD z scores at the spine (mean, -0.34 ± 0.8; P = 0.01) in comparison to controls. Among 32 patients with mHPA, no one with Z score < -2 was recorded and ten had low-normal Z scores. Their MM and FM were comparable to controls. 

**Figure 1.**
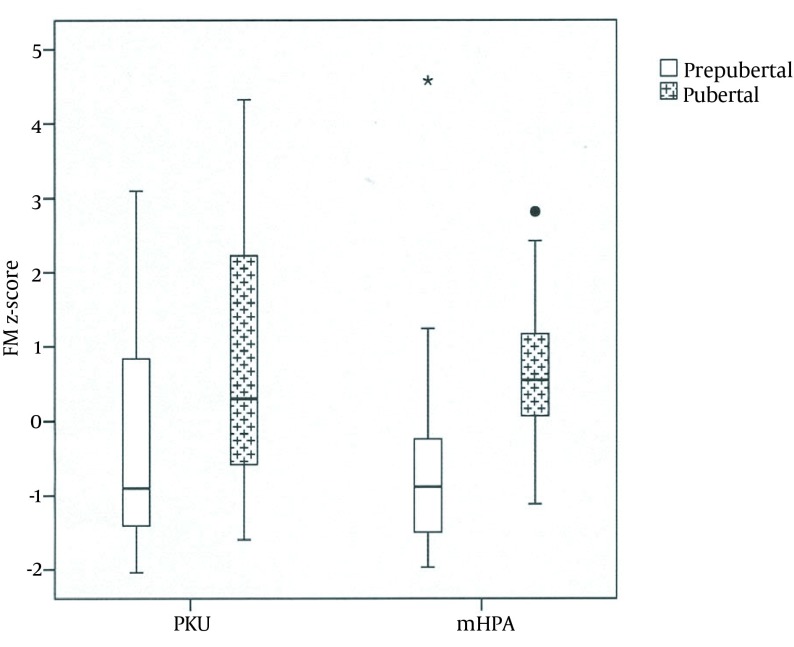
Z Scores of Fat Mass in Prepubertal and Pubertal Patients With Phenylketonuria and Mild Hyperphenylalaninemia

No significant difference was detected in body composition parameters between patient with PKU and mHPA. With regards to within-group comparisons, there was no gender impact on the results in patients of both groups; however, pubertal status was important, as it is illustrated in Table 2 In other words, in the PKU group, significant increase in FM was recorded in the adolescents in comparison to the prepubertal patients. In the patients with mHPA, there was a remarkable increase in both MM and FM during puberty; this trend was not found in prepubertal stages (Figure 1).

**Figure 2.**
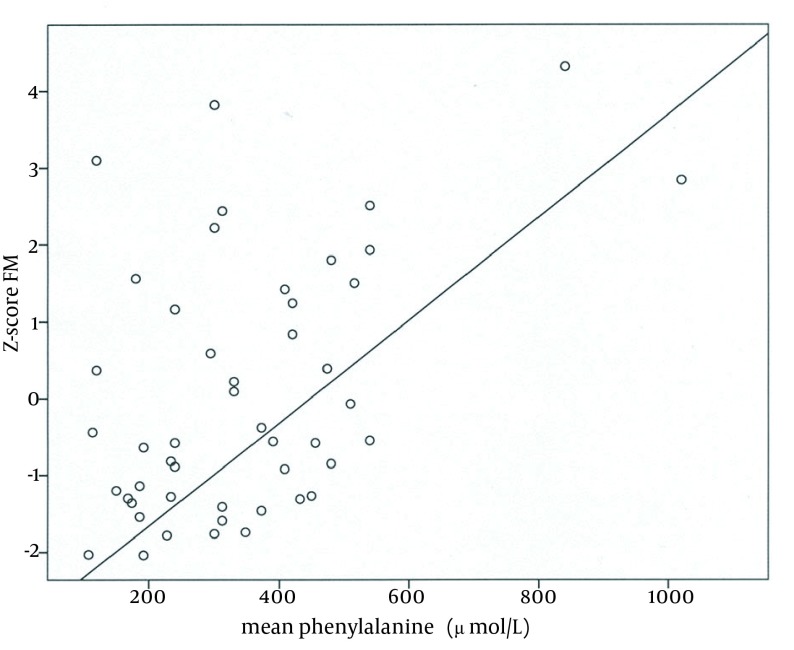
Positive Correlation Between Fat Mass and Mean Phenylalanine Levels in Patients With Phenylketonuria

The next step was to investigate possible correlations between BMD at both sites, LTM, and FM in both groups of patients as well as between mean Phe levels and body composition parameters in PKU group only; these results are illustrated in Table 3. Apparently, in both patients groups, significant positive correlations exist between BMD (at both sites) and FM. In patients with PKU, there was also a positive correlation between BMD and MM, whereas Phe level did not seem to be related to BMD. On the contrary, an association between Phe and fat content was evident (r, 0.46; P = 0.001; Figure 2). 

**Table 1:** Overview of the Study Population Profile^a,b,c^

Variable	PKU (n = 48)	mHPA (n = 32)
**Age, y**	10.9 ± 3.43	10.85 ± 3.6
**Sex**	-	-
Male	25	18
Female	23	14
**Prepubertal**	26	20
**pubertal**	22	12
**Ht (z score)**	- 0.2 ± 1.29	0.4 ± 1.08
**Wt (z score)**	0.28^d^ ± 0.91	0.28 ± 0.93
**BMI (z score)**	0.34^d^ ± 1.02	0.23 ± 0.95
**Mean Phe, μmol/L**	344 ± 178	222 ± 51.6

^a^ Abbreviations: PKU, phenylketonuria; mHPA, mild hyperphenylalaninemia; Ht, height; Wt, weight; BMI, body mass index; Phe, phenylalanine.

^b^ Data are presented as mean ± SD.

^c^ Compared to the reference population (Greek growth charts) using student’s t test.

^d^ P < 0.05.

**Table 2:** Within-Group Comparisons of Body Composition Profiles, Based on Pubertal Status^a,b,c^

Variable (Z Score)	PKU Prepubertal	PKU Pubertal	P Value	mHPA Prepubertal	mHPA Pubertal	P Value
**BMD LS**	-0.33 ± 0.9	0.16 ± 1.4	NS	-0.5 ± 0.6	0.04 ± 1.1	0.054
**BMD TB**	-0.12 ± 1	0.39 ± 1.1	NS	0.12 ± 0.7	0.52 ± 1.3	NS
**LTM**	-0.23 ± 1.1	-0.1 ± 1.3	NS	-0.65 ± 1	0.7 ± 1.1	< 0.01
**FM**	-0.45 ± 1.4	0.66 ± 1.7	0.018	-0.6 ± 1.5	0.7 ± 1	< 0.01

^a^ Abbreviations: PKU, phenylketonuria; mHPA, mild hyperphenylalaninemia; BMD, bone mineral density; NS, nonsignificant; LS, lumbar spine; TB, total body; FM, fat mass.

^b^ Student’s t-test.

^c^ The z scores are presented as mean ± SD.

**Table 3:** Pearson’s Correlations Coefficient Between Body Composition Parameters in Patients With Phenylketonuria and Mild Hyperphenylalaninemia ^a,b,c^

BMD	LTM	FM	Mean Phe
**PKU group**	-	-	-
BMD LS	0.344^d^ (0.017)	0.347^d^ (0.016)	0.21 (NS)
BMD TB	0.424^d^ (0.003)	0.4^d^ (0.004)	0.13 (NS)
**mHPA group**	-	-	-
BMD LS	0.342 (NS)	0.352^d^ (0.049)	-0.23 (NS)
BMD TB	0.285 (NS)	0.428^d^ (0.015)	-0.34 (NS)

^a^ Abbreviations: z, z score; BMD, bone mineral density; LTM, lean tissue mass; FM, fat mass; PKU, phenylketonuria; Phe, phenylalanine; mHPA, mild hyperphenylalaninemia; LS, lumbar spine; NS, nonsignificant; TB, total body.

^b^ Correlation coefficients are presented as r (P value).

^c^ The correlation was drawn between the z score of parameters.

^d^ P < 0.05.

## 5. Discussion

There is a vivid worldwide interest toward the growth and bone health in patients with inborn errors of metabolism who are under special diet regimes. This study revealed that patients with PKU were weightier in comparison to their healthy Greek counterparts. This is in accordance with other studies that yielded similar results (10-13). The MM and FM of the patients with PKU was not significantly different while their TB BMD was lower in contrast to their controls. It is well established that bone health in PKU is at risk of morbidities. Numerous studies have shown low BMD in patients with PKU, which is attributed to different predisposing factors including low protein as well as fatty acid intake (14, 15) and the disorder itself through a direct toxic effect of excessive Phe on bones (16). Our patients had lower TB BMD than controls. TB measurement represents cortical bone status and is influenced mostly by nutrition. A study in young adults with PKU reported a lower peak bone mass than controls (17) and another group of researchers reported low cortical thickness, with quantitative ultrasonography (16). This suboptimal bone profile does not necessarily lead to increased fracture rate; however, data on this field are contradictory (18). On the other hand, patients with mHPA had normal growth as reported previously (16) and their MM and FM were not different from the reference population; however, LS BMD was decreased in comparison to controls. To our knowledge, there was no other study concerning BMD assessment of patients with mHPA by DXA. There was only one study on bone quality, conducted with a different method (quantitative ultrasonography), which reports no significant abnormalities (16). Considering that lumbar spine measurements refer to the evaluation of trabecular bone, which shows very active bone turnover, the finding that in mHPA patients it is the spine that is affected the most, is intriguing and needs further investigation, ideally with parallel evaluation of metabolic bone markers. It is noteworthy that these patients can act as models in the research of the effect of chronic exposure to high Phe levels on bone, as they are on a free diet. MM is less investigated in disorders of Phe metabolism. Adamczyk et al. (15) reported normal MM only in those who adhered to their diet. Other studies reported no differences in MM, irrespective of diet compliance (19, 20). These conflicting results cannot be used in isolation to determine MM. Other parameters, such as creatinine excretion in urine, grip force, or forearm muscle area assessed by peripheral quantitative computed tomography should also be evaluated along with body composition measurements, to provide more information on the actual muscular status of each patient (21). 

Obesity is another issue that deserves further research in PKU and mHPA. Increased FM in patients with PKU in comparison to controls is reported (12). Similar to our study, other studies found no difference in FM (14, 20). Apparently, different exercise levels and diet strategies account for these results. In addition, it should be kept in mind that Greek children and adolescents are among the most obese ones in Europe (22, 23). This could contribute to the fact that our patients were not more obese than controls and highlights the importance of using native reference populations for comparison. In both groups, a tendency towards increased adiposity during puberty was recorded, which is a period when FM increases substantially as is evident by using air-displacement plethysmography (12); however, in those teenagers with PKU and poor dietary compliance, this tendency was more pronounced, which was confirmatory to our initial clinical impression and working hypothesis. The management protocol definitely play a significant role (11,24). Studies looking into possible association of Phe levels with biochemical markers of adiposity (eg, adiponectin, leptin, etc) are warranted to guide further management. Basic laboratory profile was normal in both groups, which was in accordance with other works (17). A positive correlations were recorded in both groups between BMD, MM, and FM while such an association could not be established between Phe levels and BMD, which was in accordance with the existing evidence (25). The observed positive correlation between Phe levels and FM is intriguing as was reported previously (14, 26). Another useful remark is the weakness of BMI as an index of adiposity; although patients with PKU had higher BMI, their FM was comparable to controls. Therefore, for body composition analysis, FMI (FM index, FM g/1000/Ht square [m²]) is preferable to BMI (27). Unfortunately, appropriate age- and sex-specific FMI cut-offs for children have not been established yet. Despite the agreement of our findings with most of the available evidences, certain limitations exist, most of which result from poor financial resources and the short study period. Ideally, a study combining BMD with metabolic bone markers, grip strength, or scoring of the level of physical activity or screen time (as a reflection of sedentary lifestyle) would offer valuable information. In fact, a new study should be planned with these parameters in mind. In conclusion, since bone and fat seem to be affected in Phe metabolism disorders, every effort should be made towards better adherence to the proposed diet and a healthier lifestyle, especially during adolescence. In addition, regular follow-up of bone health and body composition has much to offer, as a way of monitoring nutritional status and quality of growth. More studies are needed to reveal the possible interaction of Phe with bone and adipose tissue.
